# The Effect of Pandemic-Related Economic Disruption on Young Adolescents in Ireland

**DOI:** 10.3390/children9071037

**Published:** 2022-07-12

**Authors:** Emer Smyth, Aisling Murray

**Affiliations:** Economic and Social Research Institute, D02 K138 Dublin, Ireland; aisling.murray@esri.ie

**Keywords:** COVID-19, pandemic, economic resources, financial strain, child well-being, family relationships, homeschooling

## Abstract

The sudden health and economic crisis brought about by the COVID-19 pandemic affords an opportunity to examine the impact of economic disruption to children and families. Any negative effects on the well-being of children are important to consider in relation to both short- and long-term outcomes. Using pre-pandemic and mid-pandemic waves of the longitudinal Growing Up in Ireland study, we examined whether the impact of economic disruption was equivalent for families who were (or were not) financially vulnerable pre-pandemic. We then investigated whether economic disruption was associated with a negative effect on the emotional well-being of 12-year-olds, and if there was evidence for such a negative effect being mediated through a lack of material resources or strain on family dynamics. Our results indicated that middle-income rather than lowest-income families experienced the most economic disruption, likely reflecting the sector-specific nature of business closures in the pandemic. Families who were financially vulnerable pre-pandemic were less likely to have had suitable resources for homeschooling. Both falls in income and strain in family relationships, such as arguing more with their parents, were associated with poorer scores on a measure of the child’s emotional well-being. The emergency income support payment introduced at the start of the pandemic appeared to have a protective effect on the association between family income loss and child well-being, which has wider implications for policy on child poverty.

## 1. Introduction

The COVID-19 pandemic was, and—at time of writing—continues to be, a far-reaching event that affected virtually everyone on the planet in some way. Public health measures introduced to save lives included the mass closures of workplaces, schools and other services with unprecedented scale and suddenness. Few adults (in Ireland at least) would have had any similar previous experience on which to draw, and so one must assume that children and young people were even less prepared for the disruption to almost every aspect of their normal routine. Given the sudden and unique nature of the pandemic, there is, therefore, a significant information gap around the impact of such disruption and which subgroups are most affected. Evidence from nationally representative samples, particularly those with pre-pandemic benchmarks, will be important for informing policy responses in the post-pandemic period. The Growing Up in Ireland study (GUI) is one such study, comprising two cohorts: Cohort ’98 and Cohort ’08 (aged 22 and 12 years, respectively, in December 2020). 

In this paper, we focus on how children’s mental well-being was affected (or not) by the economic impact of the pandemic on their parent(s) using data from Cohort ’08 of the GUI study. We hypothesise two potential non-exclusive mechanisms for such a relationship. The first is the more or less direct impact of reduced income (either recent or longstanding), where the child does not have access to resources typically available in wealthier households, such as educational or entertainment resources. For example, a UK study by Metherell et al. [1] noted a link between ‘digital exclusion’—as measured by being without access to a computer and/or a good internet connection—and poorer socio–emotional well-being among teens, especially in the earlier stages of the pandemic (which later declined over time). Even if children had been similarly deprived before the pandemic, lockdown and school closures likely made the absence of resources (or overcrowding in the home) more acute. 

The second mechanism is more indirect: the negative impact of economic disruption on the mental well-being of the parents, which, in turn, upsets the children. This could be because stressed parents are less able to maintain high-quality interactions with their children or simply that the child observes their parents’ distress and worries about them. The former is essentially the ‘family strain model’, which was explored by Nixon et al. [2] with the GUI Cohort ’08 at age 3, in the context of the Great Recession. Overall that report found economic strain was associated with parental depressive symptoms, which in turn affected marital satisfaction and parent–child interactions; however, the link with actual child outcomes was weak. 

### 1.1. Evidence for Economic Inequalities in the Pandemic

Drastic public health measures inevitably have economic consequences for families, in addition to disruptions to normal routines. In Ireland, as elsewhere, whole sectors—especially hospitality—were shut down literally overnight in the first ‘lockdown’ of March 2020. A survey of adults by the Irish office of national statistics (Central Statistics Office, CSO) as part of a COVID module in the Labour Force Survey, released in May 2020 (just after the start of the first lockdown), showed that just under half of the adult population said their employment had been impacted by the pandemic [3], rising to two-thirds of those in the 35–44 year group (the likely age of parents of 12-year-olds, see later). One-third of those affected were temporarily laid off and 14% lost their jobs. Those who could, worked from home, but not every role could be adapted in that way, even if an individual was not employed in a business that had to shut down entirely. The same CSO survey found that one-third of impacted employees started working from home (over 40% of those in the 35–44 age group), but 20% were in a role that could not be done remotely. A later CSO survey of businesses for the 2020 calendar year [4,5] found that the ‘professional and IT’ sector had the highest percentage of employees working remotely (79%) and were the least likely to have had to close at some point during the year (27%); whereas the ‘accommodation and food services’ sector was the least likely to have staff working remotely (8%) and the most likely to have closed at least once (90%). Therefore, whereas everyone faced the public health threat posed by the virus to some degree, the economic disruption was hugely variable. Blundell et al. [6] note this sectoral inequality as one of the defining features of the economic impact of the pandemic compared to a ‘normal’ recession, the other being the simultaneous disruption to almost every other area of life—such as education, socialising and travel—that created further economic disruption in themselves.

The potential for economic inequalities as a result of the pandemic also has (at least) two dimensions. First, there is the economic ‘shock’ caused by sudden job and/or income loss as a direct result of business closures and curtailment in lockdown. As we have already seen, there was huge sectoral variability in the likelihood of experiencing such a shock. However, data from the UK (Understanding Society) indicate that families where parents had low levels of education, insecure contracts or were self-employed were also at greater risk of a sudden drop in or cessation of earnings [7]. Analysis of Irish data on ‘who can work from home in the pandemic’, based on pre-COVID patterns of home-working and sector of employment in the Irish Labour Force Survey, showed that workers who were over 30, men, Irish nationals, in full-time contracts, in higher-paid occupations, and not a one-parent family were more likely to be able to work from home [8]: in particular, they noted the over-representation of lone parents among essential workers and the childcare difficulties faced by such individuals given the school and childcare closures during lockdown. Analysis of UK data by Cheng et al. [9], comparing working parents with other workers—and using COVID and pre-COVID waves of data from the Understanding Society survey—found working parents reported more financial insecurity, worse mental health, were more pessimistic about their financial future, and were more likely to have received financial support in the COVID survey. Cheng et al. noted that workers and working parents had not differed on the last two measures (financial pessimism and financial support) in the pre-COVID wave [9].

Secondly, in terms of economic inequalities, is the differential impact of the disruption depending on what resources individuals and families had to either cushion a sudden income shock or cope with the closure of services (such as schools) that they would normally avail of. For example, a family already financially dependent on social welfare would not have experienced a cut in that payment, but they would have been less able to supplement their child’s education with other paid tuition—or even provide a computer for free online lessons—when schools closed. Blundell et al. [6] summarise figures from the Department of Education in the UK that indicate that, while all children experienced a drop in learning hours during the school closures there, the drop was steeper for children in the poorest quintile than the wealthiest, and the latter also spent more time in interactive activities thought to be more beneficial to learning. Further, in the UK, analysis of COVID-survey data from Understanding Society indicated that children in families where the father’s income had dropped to zero in the pandemic were less likely to get additional paid-for learning resources [7], but there was an increase in the time parents spent with them on schoolwork.

### 1.2. Recent Research on the Pandemic’s Effects on Well-Being

The findings from recent research on the impact of COVID on well-being have been mostly, but not entirely, negative. Some findings from the UK suggested that parents’ perceived relationship with their children actually improved over lockdown—at least initially: analysis of data collected in May/June 2020 (as part of the Understanding Society COVID study) by the Centre for Population Change [10] described 70% of parents saying their relationship with children had stayed the same and 26% improved, with just 4% saying it had gotten worse. However, one-parent families, those spending long hours in childcare, who reported a difficult financial situation and/or did not have enough space to work from home were among the minority who said the parent–child relationship had deteriorated. Several reports have commented on pandemic-related increases in mental health problems among adults. For example, the Born in Bradford study observed increases in the prevalence of moderate/severe depression and anxiety from pre-COVID to COVID (March–June 2020) among mothers in its cohort [11]. In that study, clinically important increases in symptoms were associated with, among other things, insecurity around food, finance and housing. 

In the Irish case, comparing pre-COVID and mid-COVID indicators of well-being among adults (not necessarily parents) surveyed by the CSO [12] showed a decline in most measures in November 2020 as well as in April 2020. Overall life satisfaction, satisfaction with personal relationships and the percentage feeling happy most or all of the time all declined during COVID waves, whereas the percentage feeling downhearted/depressed or nervous increased. The number of adults feeling downhearted/depressed most or all of the time almost doubled between April 2020 (5.5%) and November 2020 (11.5%). 

Several studies also point to widespread mental health concerns among adolescents during the pandemic. The Scottish ‘Teen COVID Life’ surveys of 2020, before and after school closures, indicated more stress, lower mood and greater loneliness among girls than boys—and in the second survey almost half of adolescents felt that COVID had an overall negative effect on their lives, with nearly 20% reporting low satisfaction with life and school [13,14]. These ‘Teen COVID Life’ surveys were volunteer samples with some participants completing both waves. Longitudinally—using the youth panel of the UK’s Understanding Society study—Hu and Qian [15] observed a worsening of self-reported emotional symptoms and problems with peers among those aged 10–16 years but an improvement in conduct problems (comparing before and after the start of the pandemic). The same authors noted that loss of household income did not seem to be associated with change in adolescent mental health (so was not included in the model), but there was a greater decline for those who were in low-income families pre-pandemic. In contrast, using England’s Mental Health of Children and Young People study, Newlove-Delgado et al. [16] reported not just an increase in the rate of 5–16-year-olds with ‘probable mental health problems’ from 10.8% (in 2017) to 16% in July 2020, but also that “children with probable mental health problems were more than twice as likely to live in households newly falling behind with their bills, rent, or mortgage payments compared with those whose families were able to pay their bills” (p. 353). A recent (February 2022) survey of school-aged children and young people in Ireland (recruited through invitations to schools) reported that, “during or because of the pandemic”, ‘a lot’ or ‘all of the time’ they had felt lonely (29%), unhappy (29%), worried (40%), angry (21%) or bored (65%). However, some also reported experiencing positive emotions ‘a lot’ or ‘all of the time’: happy (48%), hopeful (26%), satisfied (24%) or safe/secure (56%) [17].

### 1.3. The Current Study

As noted earlier, this work uses data from the younger cohort (’08) of the Growing Up in Ireland (GUI) study who would have been around 12 years old in 2020, the first year of the pandemic. Insights into the possible impact of economic upheaval on child well-being comes from earlier work with both this cohort (then aged 9 months—3 years) and the older cohort (’98) of GUI. The children of Cohort ‘98 would have been a similar age when the Great Recession of 2008–2013 took hold as those in Cohort ’08 were at the start of the pandemic in 2020. Watson et al. [18] exploited the timing of the first two waves of GUI at just before and during the Great Recession to examine the effect of economic vulnerability on child socio–emotional well-being. For both cohorts, being in a family that was ‘economically vulnerable’ during both waves was associated with a markedly higher risk of socio–emotional difficulties (13%) compared to being vulnerable at one of the two waves (8%) or neither (4%). However, after controls for other family characteristics associated with risk of economic vulnerability, only the contrast between both waves and neither wave was statistically significant. This finding in relation to the impact of economic disruption associated with or predating the Great Recession suggests that, this time around, children who were in already financially distressed families will be more vulnerable to the mental health impacts of further decline than those whose financial downturn is more recent. However, as already noted, the economic disruption arising from the Great Recession was not either as sudden or concomitant with widespread disruption to other areas of children’s lives as the pandemic. The government policy response was also quite different: tough austerity measures were used to counter the former, but the pandemic response was to increase government expenditure in an effort to support family incomes and keep businesses afloat [19].

The multiple-disruption feature of the pandemic does raise some difficulties in separating economic effects on children’s well-being from other risk factors such as isolation due to reduced contact with family and friends, and the ‘existential crisis’ posed by the virus. There are some mitigating factors to this dilemma, however. In the first instance, the lockdown restrictions and the pervasive nature of the virus affected all children, although it would be disingenuous to claim that the effect was universal and equal. Secondly, the nature of the information collected in the GUI COVID survey used in this analysis allows us to control for other known or suspected risk factors for poorer mental well-being, such as gender, changes in sports/physical exercise and the consumption of junk food. Thirdly, the longitudinal nature of the survey means that we can control not just for pre-pandemic levels of income but also for the child’s socio–emotional well-being at age 9 years.

A further variable of interest in this analysis is the timing of the pandemic with this cohort’s transition from primary to secondary school (which occurs around age 12 to 13 years in Ireland). As it happens, a portion of this cohort abruptly finished their last year of primary school in March 2020 and, when schools reopened in September 2020, went straight into their first year of secondary school. These children thus made an important transition without the potential benefit of preparation in the last quarter of primary school and started in brand new school system in the context of COVID. The rest of the cohort returned to primary school in September 2020 for what would likely be their final year. The GUI data allow us to control for whether children had recently made the transition to secondary school in examining their mental well-being.

### 1.4. Rationale and Hypotheses

The impact of economic disruption to parents on the well-being of their children is a key feature for understanding future development and trajectories for this cohort. The timing for GUI Cohort ’08 is of particular relevance given the important transition to secondary education at this time, and due to the stage of maturity of 12-year-olds—who might be more aware of the impact on parents than at earlier ages. So, the first reason for examining the short-term effects of the pandemic on children relates to their importance for the longer-term outcomes of the cohort. Secondly, the suddenness of the disruption and the absolute numbers of families affected provides an (almost) unique opportunity to examine the effect of sudden job and income loss without the usual levels of ‘noise’ in relation to the timing and reasons for that loss. Thirdly, because the GUI COVID survey collected data directly from children, we can include the child’s own perceptions of the situation with the factual information on the economic situation from their parents.

This leads to three primary hypotheses about the relationship between economic resources, family dynamics and child well-being during the pandemic:

**Hypothesis** **1.** *Families who were financially vulnerable prior to the pandemic will be more likely to report economic disruptions such as financial strain and difficulty providing homeschooling resources during the pandemic*.

**Hypothesis** **2.** *Economic disruptions will negatively affect self-reported child well-being through both a lack of resources available to the child and changes in the emotional quality of the parent–child relationship*.

**Hypothesis** **3.** *The child’s well-being will be affected by other pandemic-related restrictions on contact and activities in place at the time of the survey (December 2020)*.

### 1.5. Background Information on the Pandemic Situation in Ireland

It is important to understand the timing of the survey in relation to the waves of COVID-19 and associated public health restrictions. As with most other countries, Ireland entered a strict lockdown, including the closure of schools and instructions to work from home except for essential services, in March 2020. There was some easing of restrictions that summer, and schools reopened for the first time for the new school year in September. There was a second wave of the virus in October and strong restrictions on travel and socialising were reintroduced that month, but, unlike the previous lockdown, schools remained open. As the GUI COVID survey was launched in December 2020, some restrictions were being lifted and further easing was expected in the run up to Christmas. The announcement of a vaccine breakthrough had come a couple of weeks previously. 

A CSO survey of Irish adults conducted in November 2020 [20] on sentiments around the upcoming Christmas period found that three-quarters of respondents were worried about not being able to mix with other households or see friends or family. While most respondents (89%) expected to spend the same or less than the previous Christmas, households with children were more likely to expect spending more (16%) than multi-adult households without children (7%). People in rental accommodation (with or without children) were more likely to be worried about their inability to afford Christmas presents than those in owner-occupied accommodation (23% versus 8%).

The extent to which pandemic restrictions operate as an ‘economic shock’ to family living conditions will depend on the nature of COVID-related economic supports introduced. In Ireland, the Pandemic Unemployment Payment (PUP) was introduced on 13 March 2020 for those who lost their jobs as a result of the pandemic, initially at a rate of €203 per week (comparable with the maximum personal rate of existing unemployment payments). The PUP was raised to €350 per week from 24 March, thus providing those affected with relatively high levels of income replacement [21,22]. It was paid at a flat rate of €350 per week for a number of months before the rate of payment was tied more closely to previous earnings. In December 2020, when the GUI COVID-19 survey was carried out, there were four rates of payment, ranging from €203 to €350 per week. In contrast to existing unemployment supports, such as Jobseeker’s Benefit and Jobseeker’s Allowance, there was no requirement to have sufficient social insurance contributions and/or to pass an income means test. The Employment Wage Subsidy Scheme (EWSS), previously the Temporary Wage Subsidy Scheme (TWSS), allowed workers to receive government support directly through their employer’s payroll, thereby maintaining the firm–worker link [21]. By the end of the first full month of the pandemic (April 2020), 620,000 individuals were claiming the PUP, and 43,000 employers with 427,400 employees were registered for the TWSS. Together, this represented 40 percent of all those employed in Q4 of 2019 [21]. Another feature of the pandemic response in Ireland was a moratorium on rent increases and evictions between March 2020 and August 2020, and thereafter a rent freeze for tenants financially impacted by the pandemic [23]. On the basis of these measures, we expect that the income disruption caused by parental job loss will be partially cushioned by the receipt of social welfare payments. 

## 2. Materials and Methods

This article draws on a special inter-wave survey of Cohort ’08 of the Growing Up in Ireland (GUI) study. The families in Cohort ’08 were sampled from the Child Benefit register and were first surveyed when the children were nine months old, with further interviews conducted at 3, 5 and 9 years of age. In December 2020, in order to capture experiences of the pandemic, a link to an online survey was sent to the mothers and the young people themselves (then 12 years of age), and the survey, hosted by the Central Statistics Office, was available from 4–31 December 2020. The response rate was 45 percent for mothers and 38 percent for young people, with weights used to account for differential attrition across groups [24]. The analyses in this article are based on the 2947 cases where both the mother and the young person completed the survey. The Anonymised Microdata Files (AMF) are used for the analysis; these data are available from the Irish Social Science Data Archive (ISSDA). 

The focus of the article is on the influence of (changes in) economic resources during the pandemic on young people’s well-being. The Mental Health Inventory (MHI-5) is used to assess well-being (including items such as ‘how much of the time in the past four weeks… have you felt downhearted and blue’?), with higher values indicating more positive well-being. The MHI-5 was used for the first time in the COVID-19 survey as it was relatively short but well-validated internationally. In order to control for well-being prior to the pandemic, we use the Strengths and Difficulties (SDQ) total difficulties score at age 9, where higher values indicate greater difficulties. Summary statistics on all of the variables used are presented in Table 1. 

The family economic resources are captured along a number of dimensions. Household income, equivalised for household size and composition, at age 9 is divided into quintiles, with an additional category of missing information on income included in order to retain cases for analysis. In addition, financial strain is measured both at age 9 and during the pandemic based on the mother’s report of the household having ‘very great difficulty’ or ‘great difficulty’ making ends meet. The model exploring the factors associated with financial strain also takes account of household social class; this is based on the higher occupation of parents and uses a classification developed by the Central Statistics Office. An additional category, ‘non-employed’, is used for households that cannot be assigned a social class because of the absence of a recent employment history; this group is important to capture given the prevalence of jobless households in the Irish context. We also control for gender, whether the household is a lone-parent family or not and whether the family lives in an urban or rural area. 

In Ireland, as elsewhere, the pandemic resulted in very significant economic disruption. We distinguish those households where one or both parents experienced job loss (or hours/income loss) as a result of the pandemic from other families. We also examine the extent of income loss, distinguishing between those who lost ‘a lot’ or ‘a little’ from all others. Receipt of the Pandemic Unemployment Payment is included as a dummy variable to examine the potential cushioning effect of this payment on living standards and therefore well-being. 

Previous research has shown that family economic resources can constrain the materials and resources young people have for learning [25], and that this was exacerbated during the pandemic, as they were dependent on access to devices, broadband and available space to engage in remote learning [26]. To take these into account, we examine whether the young people reported that it was ‘always true’ (as opposed to ‘sometimes true’ to ‘never true’) that they had a quiet place to study and access to a computer when they needed it during the (first) period of school closures. Because two-thirds of the 12-year-olds made the transition to secondary school over the period, we take this into account given the well-established effect of the transition process on well-being [27].

The family stress model indicates that constrained resources can operate indirectly on child well-being via parental stress [28]. In order to unpack stress among children and parents, we take account of whether the young person reports feeling ‘always’ or ‘sometimes worried’ about COVID-19 and whether they have had direct experience of the virus (having had it themselves or a family member (or close contact) having had it). In addition, we take account of whether the young person reports that it is always or sometimes true that ‘I can see that my parent or parents are worried at the moment’. The extent to which strain spills over into conflict is captured by a measure of whether the young person reports arguing more than usual with their parents and (separately) with their siblings. The pandemic may have had positive effects by increasing the amount of time families spend together [29]. In order to separate out the effects of enforced time at home (because of remote working), we take account of whether one or both parents shifted to remote working (or increased their hours doing so) and also the young person’s report of whether they were spending more time with their families than before the pandemic.

The pandemic and related health restrictions resulted in considerable disruption to day-to-day activities and behaviours. We capture these by a series of questions to the 12-year-olds on the extent to which they engaged in the following more, about the same or less compared to before the pandemic: seeing friends face-to-face, taking part in sports/physical exercise, spending time outdoors, taking part in structured cultural activities (such as drama or music lessons), spending time on screen-based activities (not for school) and eating more junk food/sweets.

The main analyses focus on the effects of these sets of factors on child well-being using a series of nested regression models which control progressively for: family economic resources; educational resources and child and parent worries/concerns; and changes in involvement in social activities. 

## 3. Results

This section starts by looking at the economic circumstances experienced by families before and during the COVID-19 pandemic before assessing the impact of these experiences on mental well-being among 12-year-old young people. 

### 3.1. The Economic Impact of the Pandemic

A longitudinal perspective means that we can trace the extent of the way in which families experience financial strain reflects both the economic cycle and the onset of the pandemic. In 2008/9, on the cusp of the Great Recession, when the children were nine months old, 13 percent of the families reported difficulty or great difficulty making ends meet; this figure increased to 22 percent as the recession progressed and was 26 percent when the study children were 5 years of age, before declining subsequently [30]. The pandemic did not result in changes to *average* levels of financial strain; in fact, the percentage of mothers who report their household had (great) difficulties making ends meet was slightly lower during the pandemic than it was three years previously (11% compared with 12%). At the same time, over a third of households (36%) reported that their incomes had fallen ‘a lot’ or ‘a little’ since the start of the pandemic. This fall in income largely reflects the extent of job loss (or reduced hours/income) among the parents surveyed; in 31 percent of families, one parent experienced such employment disruption, while this applied to both parents in 5 percent of cases. Interestingly, job losses and income declines were somewhat more common for those who had been in the middle-income quintile three years previously and lowest for the highest quintile. This suggests that employment shocks in Ireland tended to be sector-specific rather than disproportionately affecting lower income or more vulnerable workers (see above). 

Table 2 looks at the factors associated with experiencing financial strain during the pandemic. Those who had experienced such strain three years previously were much more likely to remain in this category, even controlling for prior income and household social class. Exposure to financial strain was strongly related to prior occupation/social class, with much higher levels of strain found among those not in professional or managerial jobs (Model 1). Among those in employment, financial strain was particularly high among skilled manual workers. Part of this effect relates to greater job losses among this group, most likely reflecting the closure of the construction sector and much of the manufacturing sector during the period of public health restrictions. In terms of income group, those who had been in the lowest quintile had the greater likelihood of financial strain, while those who had been in the highest quintile were very unlikely to experience such strain (Model 2). Occupational effects largely operated through income level. However, even controlling for prior income, those who had been in skilled manual jobs or who had not held a job were most likely to experience strain. Households who had experienced pandemic-related job loss were much more likely to report financial strain. Interestingly, and contrary to our expectations, receipt of the Pandemic Unemployment Payment did not appear to have a protective effect against financial strain.

As discussed above, pre-existing financial strain and/or the economic shock of the pandemic could curtail the educational resources young people had for remote learning as well as the kinds of social activities in which they engaged. Those who lived in families experiencing strain when they were aged 9 were somewhat less likely to subsequently have a computer to engage in remote learning (66% compared with 74%) or to have a quiet place to study (42% compared with 49%) (Table 3). Those experiencing strain during the pandemic were less likely to have a quiet place to study (43% compared with 50%) or a computer (66% compared with 74%), while those who experienced a fall in income were less likely to have a computer (70% compared with 75%). Overall, Hypothesis One that families already economically vulnerable pre-pandemic would experience greater financial strain during the pandemic and be unable to afford the same level of educational resources for their children is supported. 

### 3.2. Economic Resources and the Young Person’s Well-Being

A series of regression models were used to analyse the extent to which economic resources were related to the young person’s well-being and the factors that may have mediated this relationship. 

Table 4 looks at the relationship between family economic resources and child well-being. Household income three years prior to the pandemic has no significant influence on well-being (Model 1). However, young people in lone-parent families, living in urban areas and in households where one or both of their parents lost their job (or had reduced hours) had significantly poorer mental health (Model 1). The effect of job loss is linked to the associated reduction in income (compare Models 1 and 2), with receipt of the Pandemic Unemployment Payment associated with a protective effect on well-being. These patterns suggest that parental job loss was primarily an economic shock rather than the broader psychological consequences of being without work affecting child well-being. Reporting persistent economic strain or strain during the pandemic are not linked to well-being once income level and income reductions are taken into account (an alternative specification (not shown here) showed no relationship between financial strain and well-being even before taking account of income level and reduction). Not surprisingly, young people who had higher levels of (parent-reported) socio–emotional well-being at age 9 had poorer well-being at age 12. The gender difference is even larger when prior SDQ is taken into account, indicating a greater decline in well-being for girls than boys, while poorer well-being among those in lone-parent families is found to be due to prior difficulties being greater. The effects of income decline and PUP receipt are robust to the inclusion of prior well-being measures. 

Table 5 adds in educational resources, the extent to which young people had experience of or were worried about COVID-19, and whether they felt that their parents were worried. Part of the effect of income declines on well-being is mediated through educational resources. Young people who reported having a quiet place to study and a computer during the period of school closures had much better well-being than those who lacked such resources. The analysis also controls for changing schools, as the pandemic coincided with the period of transition to secondary education for this age group. As indicated by previous research [27], this transition was associated with lower well-being. Personal experience of COVID-19, that is, the young person or a family member having had the virus, was related to lower well-being (but was on the margins of significance; *p* < 0.10). Being worried about the virus (either always or sometimes) had an even stronger relationship with poorer well-being. A quite large gap in well-being was evident between young people who reported seeing their parents ‘are worried at the moment’ and other young people. Once parental worries are taken into account, the negative effect of income falling reduces in size. This suggests that much of the effect of the economic shock of the pandemic affects child well-being via young people seeing their parents concerned about economic (and potentially other) issues. The first part of Hypothesis Two is therefore supported, as we see that child well-being is negatively influenced by the lack of educational resources to engage in remote learning. 

The disruption to family economic resources took place in a broader context of serious curtailments to day-to-day activities. There was little relationship between financial strain three years previously and changes in children’s social activities over the pandemic (Table 6), though this group was more likely to have reduced face-to-face contact with friends (52% compared with 44%) and to spend less time outdoors (32% compared with 25%). However, those who experienced a fall in income were slightly more likely to reduce their involvement in sports/physical exercise (40% compared with 35%), were more likely to see their friends face-to-face less (49% compared with 43%) and ate more junk food/sweets (33% compared with 27%). Those who reported financial strain in the wake of the pandemic somewhat reduced their involvement in cultural activities (65% compared with 58%), were less likely to spend more time on screens (56% compared with 61%), spent less time outdoors (33% compared with 25%) and saw their friends less (53% compared with 44%). Despite these differences, much of the changes in social activities during the pandemic appeared to hold across families with different levels of economic resources. 

Table 7 looks at the extent to which the effect of changes in family contact and relationships over the pandemic are associated with well-being; it also examines the effects of changes in social activities. Young people who reported arguing more with their parents during the pandemic tended to have poorer scores on the well-being measure, and this factor mediates some of the effects of parents being worried. Thus, parents being worried about their financial circumstances appears to lead to more interfamily tension and therefore poorer child well-being. In contrast, more frequent arguments with siblings were not significantly related to mental health. Young people may have spent more time with their family during the pandemic because of families engaging in more activities together due to restrictions in interacting with others or because parents were engaged in remote working and therefore spending more time at home. Having one (or, to some extent, both) parents working from home is found to be linked to poorer well-being, a pattern that is consistent with the increased stress reported by parents in combining remote working and homeschooling [31]. However, spending more time with family otherwise is associated with better well-being. 

The pandemic and associated public health restrictions resulted in significant disruption to young people’s social lives, though at the time of the survey, some restrictions had eased and schools had reopened. Young people who reported spending more face-to-face time with their friends tended to have better scores on the well-being measure, while well-being scores were somewhat poorer where interaction with friends was less than it had been before the pandemic. Being more involved in sports appeared to have a protective effect, while spending less time outdoors was linked to poorer well-being. Somewhat surprisingly, being less involved in cultural activities was somewhat associated with better well-being. The latter pattern may reflect the gendered nature of engagement in cultural activities and the poorer well-being found among girls on average. Eating more junk food or sweets and spending more time online were both associated with poorer mental health. Some of the observed effects of income declines and parental worries were mediated through changes in social activities, suggesting curtailment of some social participation in response to financial pressures. 

In sum, the findings support both Hypotheses Two and Three, in linking child-well-being to the effects of both changes in family relationships and restrictions on activities and social contact. 

## 4. Discussion

This article draws on Irish longitudinal data on parents and children collected before and during the COVID-19 pandemic to document the relationship between economic resources and child well-being. Like many other countries, the pandemic restrictions in Ireland resulted in a sharp economic shock for families, with significant levels of job loss and resulting income drops, albeit partially cushioned by the introduction of a new income support scheme. The contribution of the article is that it provides new insights into the extent to which any effects on children of (changes in) parental economic resources related to lacking the resources to engage in remote learning during the period of school closures and/or to the strain on family relationships caused by declining income.

International research has highlighted the way in which the pandemic has reinforced pre-existing inequalities [6]. These patterns are, at least to some extent, echoed in Ireland. In keeping with our first hypothesis, those who experienced financial strain (having difficulty making ends meet) and with lower income levels before the pandemic are significantly more likely to report such strain during the pandemic. However, the pattern of job loss tended to be greater for middle-income than for lower-income groups, reflecting the sector-specific nature of business closures. Further, in keeping with Hypothesis One, young people in households experiencing financial strain before the pandemic were more likely to lack a quiet place to study and a computer during the period of remote learning. 

In keeping with our second hypothesis, there was evidence that the economic effects of the pandemic operated both through constrained resources and through the impact on family relationships. The effect of job loss on child well-being appeared to operate via a reduction in income so that young people who were in households where income had fallen a lot or a little had significantly poorer well-being. Interestingly, family receipt of the Pandemic Unemployment Payment was associated with a protective effect on child well-being, even though it had no direct relationship with financial strain as reported by mothers. Those adolescents who lacked a quiet place to study and a computer to engage in remote learning tended to report much poorer well-being than their peers, and these resource constraints capture some of the effect of reduced household income. Income falls are also partially mediated through young people reporting more arguments with their parents, and that their parents seem worried, suggesting greater strain on relationships in these families and significant awareness among adolescents of the economic and other pressures on their parents during the pandemic. 

Nonetheless, while the economic shock of the pandemic does have a marked effect on child well-being, in keeping with Hypothesis Three, we find that young people are also responsive to the broader impact of restrictions on their day-to-day activities. Less face-to-face contact with friends, less time outdoors, increased screentime and increased consumption of junk food/sweets were all associated with poorer well-being. In contrast, there was evidence that increased time on sports/physical exercise, increased contact with friends and spending more time with family had protective effects on well-being.

A limitation of this study is, however, incomplete contemporary information on sources of stress not related to the pandemic. While the main phases of GUI fieldwork typically collect a wealth of information in interviews lasting two hours, the rapid-response and online nature of the special COVID survey meant that questions were very much focused on the pandemic. Thus, in this analysis it has not been possible to control for other contemporary factors that might also have negatively affected child well-being, such as other recent traumatic events or current experiences of bullying. As with any voluntary research, it is also unknown whether the pandemic experiences of those who chose not to complete the special COVID survey were systematically different from those who did. To some extent, however, there is scope to address both of these limitations with the next scheduled wave of GUI’s Cohort ’98 (age 13), for which fieldwork is underway at the time of this writing.

In conclusion, the economic shock of the pandemic and the co-occurrence of largescale disruption of day-to-day life are unprecedented in scale. Nonetheless, analyses of pandemic experiences provide useful insights into the way in which economic resources affect child well-being. Longer-term economic circumstances constrain the resources for adapting to change (for example, remote learning), while sudden income falls contribute to increased worry on the part of parents and children as well as more frequent conflict. Interestingly, at least in adolescence, income support provision is found to have a protective effect on child well-being, highlighting the importance of wider poverty reduction policy for child outcomes. Furthermore, this work indicates that children in families from lower incomes and/or where there was significant economic disruption because of the pandemic are likely to need extra support both to overcome missed opportunities in relation to both formal and informal learning and to counter potentially longer-reaching setbacks to their emotional well-being. It will be important to follow the GUI cohort in the years ahead to judge the success or otherwise of both socio–emotional and economic recovery. 

## Figures and Tables

**Table 1:** Descriptive statistics.

	%
*Socio–emotional well-being*	
Mental Health Inventory (MHI-5)	74.1 (mean); 16.1 (SD)
Strengths and Difficulties Total Difficulties	7.5 (mean); 5.4 (SD)
*Family background characteristics*	
Female	48.7
Income at 9 years:	
Quintile 1	17.5
Quintile 2	17.1
Quintile 3	16.7
Quintile 4	16.4
Quintile 5	15.7
Missing	16.6
Household social class at 9 years:	
Professional	12.8
Managerial	34.1
Other non-manual	17.7
Skilled manual	13.7
Semi/unskilled manual	11.1
Non-employed	10.6
Lone-parent family	18.1
Lives in urban area	40.1
*Changes in economic resources*	
One or two parents lost job during pandemic	36.2
Received PUP	31.8
Financial strain at 9	11.7
Financial strain during pandemic	10.7
Income fell a lot during pandemic	11.7
Income fell a little during pandemic	23.8
*Educational resources*	
Quiet place to study (always true)	49.0
Computer to engage in remote learning (always true)	73.3
Changed school during pandemic	65.9
*Impact of the pandemic*	
Self or family had COVID-19	8.4
Always worried about the virus	36.3
Sometimes worried about the virus	48.7
Parents worried—always	11.2
Parents worried—sometimes	50.3
Seeing family more than before the pandemic	60.8
One parent engaged in remote working	35.3
Both parents engaged in remote working	14.0
Argue more with parents	43.4
Argue more with siblings	53.8
Involved in sports more (compared to pre-pandemic)	18.2
Involved in sports less	36.7
Involved in structured cultural activities less	57.0
See friends face-to-face less	44.9
See friends face-to-face more	17.2
Eat junk food/sweets more	28.9
More informal screentime	60.4
Spend less time outdoors	26.1
Spend more time outdoors	28.5
N (unweighted)	2947

**Table 2:** Logistic regression models of the relationship between family characteristics and experiences of financial strain during the pandemic (odds ratios).

Independent Variables	Model 1	Model 2	Model 3
Constant	0.025 ***	0.128 ***	0.077 ***
Financial strain pre-pandemic (Ref. Not under strain)	4.837 ***	3.866 ***	3.838 ***
*Income at 9 years:* Quintile 2		0.432 ***	0.398 ***
Quintile 3		0.367 ***	0.353 ***
Quintile 4		0.140 ***	0.132 ***
Quintile 5		0.045 ***	0.046 ***
Missing (Ref.: Quintile 1—lowest)		0.420 ***	0.390 ***
*Household social class at 9 years:*			
Managerial	1.600	1.116	1.112
Other non-manual	2.905 **	1.527	1.402
Skilled manual	6.768 ***	2.701 **	2.303 *
Semi/unskilled manual	4.597 ***	1.872	1.689
Non-employed (Ref. Professional)	8.949 ***	2.597 *	2.470 *
One or both parents lost job (Ref.: No job loss)			1.391 ***
Received PUP ^1^ (Ref.: No receipt of PUP)			0.917
Adjusted R^2^	19.3	25.2	26.6

^1^ PUP—Pandemic Unemployment Payment; *** *p* < 0.001, ** *p* < 0.01, * *p* < 0.05.

**Table 3:** Effect of the pandemic on children’s (self-reported) access to educational resources by experience of household financial strain before the pandemic, experience of financial strain during the pandemic and parental job loss.

	Before Pandemic		During Pandemic		Because of Pandemic	
	Strain (%)	No Strain (%)	Strain (%)	No Strain (%)	Income Fall (%)	No Income Fall (%)
Quiet place to study	41.6	49.3 **	42.7	49.6 *	50.2	48.3
Computer for remote learning (Ref. Not under strain)	65.9	74.2 **	65.9	74.2 **	69.6	75.3 ***

*** *p* < 0.001, ** *p* < 0.01, * *p* < 0.05.

**Table 4:** Regression models of the effect of family economic resources on child well-being.

Independent Variables	Model 1	Model 2	Model 3	Model 4
Constant	79.079	79.550	79.815	84.820
Female (Ref. Male)	−4.672 ***	−4.821 ***	−4.822 ***	−5.935 ***
*Income at 9 years:* Quintile 2	0.574	0.213	0.016	−0.142
Quintile 3	−0.711	−0.860	−1.094	−1.239
Quintile 4	−0.561	−0.731	−1.017	−1.751 ±
Quintile 5	−1.051	−0.956	−1.253	−2.064 ±
Missing (Ref. Quintile 1)	2.689 *	2.614 *	2.391 ±	1.664
Lone parent family (Ref. Two parents)	−2.327 **	−2.312 **	−2.213 **	−1.131
Urban location (Ref. Rural)	−3.862 ***	−3.923 ***	−3.910 ***	−3.376 ***
One or both parents lost job (Ref. No job loss)	−1.764 **	−1.306	−1.366	−1.183
*Change in income:*				
Income fell a lot		−3.765 **	−3.513 **	−3.097 **
Income fell a little (Ref. No income fall)		−3.496 ***	−3.421 ***	−3.562 ***
Received PUP ^1^ (Ref. No PUP receipt)		2.612 **	2.620 **	3.045 ***
*Financial strain:*				
Financial strain pre- and during pandemic			−1.807	−0.932
Financial strain during pandemic (Ref. No strain)			−0.560	−0.074
SDQ total difficulties at 9 (continuous)				−0.636 ***
Adjusted R^2^	4.8	5.1	5.1	9.3

^1^ PUP—Pandemic Unemployment Payment; *** *p* < 0.001, ** *p* < 0.01, * *p* < 0.05, ± *p* < 0.10.

**Table 5:** Regression models of the effect of educational resources, child and parental worries on child well-being.

Independent Variables	Model 1	Model 2	Model 3
One or both parents lost job (Ref. No job loss)	−1.363	−1.445 ±	−1.361
*Change in income:*			
Income fell a lot	−2.385 *	−2.297 *	−1.859 ±
Income fell a little (Ref. No income fall)	−2.922 ***	−3.152 ***	−2.758 ***
Received PUP ^1^ (Ref. No PUP receipt)	2.759 **	3.089 ***	2.830 ***
Has quiet place to study	5.226 ***	5.363 ***	5.208 ***
Has computer for remote learning	4.909 ***	4.772 ***	4.553 ***
Changed school over pandemic	−3.875 ***	−3.475 ***	−3.111 ***
Self or family had COVID-19		−1.984 ±	−1.843 ±
*Worried about virus:* Always		−7.709 ***	−5.098 ***
Sometimes (Ref. Never)		−3.168 ***	−2.073 **
*Sees that parents are worried:* Always			−7.328 ***
Sometimes (Ref. Never)			−4.195 ***
Adjusted R^2^	15.5	18.3	20.2

^1^ PUP—Pandemic Unemployment Payment. Note: this model controls for all of the variables included in Table 1; *** *p* < 0.001, ** *p* < 0.01, * *p* < 0.05, ± *p* < 0.10.

**Table 6:** Effect of the pandemic on children’s (self-reported) changes in activities by experience of household financial strain before the pandemic, experience of financial strain during the pandemic and parental job loss.

	Before Pandemic		During Pandemic		Because of Pandemic	
	Strain (%)	No strain (%)	Strain (%)	No Strain (%)	Income Fall (%)	No Income Fall (%)
Changes in activities						
Less sports/physical exercise	34.9	36.8	40.3	36.4	39.6 +	35.4
Less cultural activities	62.9	58.4	64.5	58.0 +	60.0	58.0
Less face-to-face contact with friends	52.1	44.3 *	53.0	44.4 **	49.4	43.0 **
More junk food	31.6	29.0	31.3	28.9	32.6	27.3 **
More screen time	59.9	60.5	56.2	60.9 **	62.2	59.4
Less time outdoors	32.2	25.3 *	33.4	25.2 **	25.6	26.5

** *p* < 0.01, * *p* < 0.05, + *p* < 0.10.

**Table 7:** Regression models of the effect of interaction with family and engagement in social activities on child well-being.

Independent Variables	Model 1	Model 2
One or both parents lost job (Ref. No job loss)	−1.430 ±	−1.654 *
*Change in income:*		
Income fell a lot	−2.333 *	−2.089 *
Income fell a little (Ref. No income fall)	−2.506 **	−2.562 **
Received PUP ^1^ (Ref. No PUP receipt)	2.917 ***	3.051 ***
*Sees that parents are worried:* Always	−6.034 ***	−5.315 ***
Sometimes (Ref. Never)	−3.531 ***	−3.314 ***
Argue with parents more	−7.373 ***	−6.890 ***
Argue with siblings more	−0.562	−0.457
See family more	1.527 **	2.223 ***
*Parental remote working:*		
One parent working remotely	−2.827 ***	−2.909 ***
Both parents working remotely (Ref. Neither working remotely)	−1.705	−2.021 ±
*Sports activities:* More		−1.548 *
Less (Ref. About the same)		−0.960
Less cultural activities (Ref. More/about the same)		0.953 ±
*Face-to-face contact with friends:* More		1.706 *
Less (Ref. About the same)		−1.182 ±
More junk food/sweets (Ref. Less/about the same)		−1.464 *
More informal screentime (Ref. Less/about the same)		−2.458 ***
*Time outdoors:* More		−0.178
Less (Ref. About the same)		−2.936 ***
Adjusted R^2^	25.5	27.6

^1^ PUP—Pandemic Unemployment Payment. Note: this model controls for all of the variables included in Table 1 and Table 2; *** *p* < 0.001, ** *p* < 0.01, * *p* < 0.05, ± *p* < 0.10.

## Data Availability

This paper uses archived data from two waves of the Growing Up in Ireland study, which are available through ISSDA. The relevant dataset citations are: 1. The Economic and Social Research Institute (ESRI). (2019). *Growing up in Ireland Cohort ’08 (Infant Cohort) Wave 5 at 9 years, 2017/2018*. [dataset]. Version 1. Irish Social Science Data Archive. SN: 0019-0. URL www.ucd.ie/issda/data/guiinfant/guiinfantwave5 (accessed on 1 April 2022) 2. The Economic and Social Research Institute (ESRI), (2021). *Growing up in Ireland—COVID-19 Web Survey 2020*. [dataset]. Version 1. Irish Social Science Data Archive. SN: 0075-00. URL www.ucd.ie/issda/data/guicovid19/ (accessed on 1 April 2022).
